# Robust Network Topologies for Generating Switch-Like Cellular Responses

**DOI:** 10.1371/journal.pcbi.1002085

**Published:** 2011-06-23

**Authors:** Najaf A. Shah, Casim A. Sarkar

**Affiliations:** 1 Graduate Group in Genomics and Computational Biology, School of Medicine, University of Pennsylvania, Philadelphia, Pennsylvania, United States of America; 2 Departments of Bioengineering and Chemical & Biomolecular Engineering, School of Engineering and Applied Sciences, University of Pennsylvania, Philadelphia, Pennsylvania, United States of America; North Carolina State University, United States of America

## Abstract

Signaling networks that convert graded stimuli into binary, all-or-none cellular responses are critical in processes ranging from cell-cycle control to lineage commitment. To exhaustively enumerate topologies that exhibit this switch-like behavior, we simulated all possible two- and three-component networks on random parameter sets, and assessed the resulting response profiles for both steepness (ultrasensitivity) and extent of memory (bistability). Simulations were used to study purely enzymatic networks, purely transcriptional networks, and hybrid enzymatic/transcriptional networks, and the topologies in each class were rank ordered by parametric robustness (i.e., the percentage of applied parameter sets exhibiting ultrasensitivity or bistability). Results reveal that the distribution of network robustness is highly skewed, with the most robust topologies clustering into a small number of motifs. Hybrid networks are the most robust in generating ultrasensitivity (up to 28%) and bistability (up to 18%); strikingly, a purely transcriptional framework is the most fragile in generating either ultrasensitive (up to 3%) or bistable (up to 1%) responses. The disparity in robustness among the network classes is due in part to zero-order ultrasensitivity, an enzyme-specific phenomenon, which repeatedly emerges as a particularly robust mechanism for generating nonlinearity and can act as a building block for switch-like responses. We also highlight experimentally studied examples of topologies enabling switching behavior, in both native and synthetic systems, that rank highly in our simulations. This unbiased approach for identifying topologies capable of a given response may be useful in discovering new natural motifs and in designing robust synthetic gene networks.

## Author Summary

Biomolecular signaling networks enable cells to mediate responses to extracellular and intracellular stimuli and are hence crucial for the functioning of all organisms. Such networks do not merely forward information, but perform signal processing: specific modules have evolved to produce complex, dynamic behaviors from input cues. Switching, or the conversion of a graded stimulus into a binary, all-or-none response, is a ubiquitous behavior that regulates critical processes ranging from cell division to stem cell differentiation. While a number of switch-generating networks have been identified, a comprehensive understanding of network architectures that can yield switch-like behavior remains elusive. In this work, we assessed the entire space of minimal networks to identify architectures that can not only exhibit switching behavior but can do so robustly in the dynamic and noisy cellular environment. Our results reveal that these robust networks fit into a small number of topological motifs. Furthermore, network composition (i.e., whether a signaling component functions as an enzyme or a transcription factor) can dramatically impact robustness in generating switching behavior. Topologies presented in this work can be used to identify additional circuits in nature that may exhibit switching behavior and suggest design strategies for engineering switching behavior in synthetic circuits.

## Introduction

Signaling networks enable cells to process information from their surroundings by eliciting temporally and spatially precise responses to environmental cues. The complex and highly interconnected biomolecular interaction networks regulating signal transmission establish connections between specific molecular effectors and hence delineate pathways through which extrinsic and intrinsic cues integrate to elicit cellular responses [1], [2]. However, it is not always apparent what minimal signaling motif is both necessary and sufficient for robustly achieving a specific behavior.

**Figure 1:**
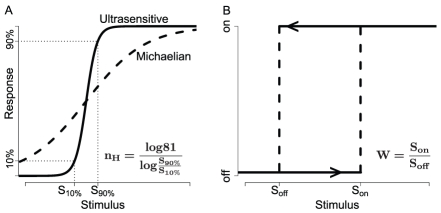
Switch-like behavior. **A.** A typical Michaelian system (*n_H_* = 1) requires an 81-fold increase in stimulus to increase the response from 10% to 90% of the maximum (i.e., *S_90%_/S_10%_* = 81) while an ultrasensitive response is more abrupt. **B.** Once triggered into the high, or ‘on’, state (*S>S_on_*), a bistable system stays in that state even as the stimulus concentration is decreased, only switching ‘off’ below a lower threshold stimulus concentration (*S_off_*, which is <0 for irreversible systems).

A signaling network that converts a graded input cue into an all-or-none response is said to exhibit ‘switch-like’ behavior; switching enables the establishment of discrete states which is vital in processes such as cell proliferation and differentiation [3], [4], [5], [6]. The term switching encompasses the more formal concepts of ultrasensitivity and bistability (Fig. 1). Ultrasensitivity is an important systems-level property in cellular contexts in which a threshold concentration of stimulus triggers entry into a different cellular state while avoiding intermediate states [7], [8]. Notable examples of signaling networks exhibiting ultrasensitivity include the MAPK cascade in *Xenopus* oocytes [9], the system regulating the mating decision in yeast [4], and the circuit controlling differentiation in the *Drosophila* embryo [10]. In biological systems, ultrasensitivity can arise from several mechanisms: positive feedback [11]; cooperativity [12], which can result from multimerization [13]; distribuive multi-site activation, in which a substrate is released from an enzyme after each activation and must re-bind before the next activation can take place [14]; and zero-order ultrasensitivity, which occurs, for example, when a kinase and phosphatase pair act on a substrate under saturating conditions [8], [15].

Although ultrasensitive systems can filter the effects of stimulus variation at concentrations far from the switching threshold, minor fluctuations in stimulus concentration near the threshold can cause the system to switch back and forth between the two states. Hence, mechanisms such as cross-antagonism and positive feedback are often employed by a cell to achieve bistability. The hysteresis, or memory effect, that arises as a consequence of bistability enables the system to tolerate stochastic fluctuations in the stimulus and the network species, and in some cases confers irreversibility, allowing the system to lose its dependence on stimulus [3], [5], [11], [16], [17], [18]. Bistability has been observed in numerous biological systems, including the *lac* operon in bacteria [19], [20], [21], the circuit regulating differentiation of erythroid and myelomonocytic lineages [6], and the circuit governing exit from quiescence in mammalian cells [22]. Bistability has also been engineered in synthetic systems using mechanisms such as cross-antagonism [13], as well as more non-intuitive mechanisms such as negative growth modulation of the host cell [23].

Previous studies have employed a combination of experiments and dynamical systems modeling to demonstrate the existence of ultrasensitivity and bistability in various signaling systems and have contributed to our knowledge of the types of network architectures that can give rise to switch-like behavior [6], [9], [10], [24], [25], [26]. However, most studies have been restricted to a few, selected network topologies and have hence explored only a small fraction of the overall space of topologies that can exhibit switch-like behavior. More importantly, the proposed topologies are not necessarily parametrically robust in exhibiting switch-like behavior, since most studies do not account for the uncertain environmental context in which networks must function. Networks that exhibit switch-like behavior only in narrow regimes of the overall biologically relevant parameter space are of diminished utility in understanding natural systems due to intrinsic and extrinsic perturbations that result in changes in species concentrations and interactions with other effectors, which are constrained at both short and evolutionary timescales by the cost-benefit tradeoff for the cell.

An unbiased, comprehensive analysis of networks that robustly generate switch-like responses in living systems would expand our understanding of the types of circuitry that enable cells to make binary decisions and assume discrete states, and hence may afford a mechanistic understanding of diseases arising out of a loss of control, such as cancer. Furthermore, such an analysis can be useful to synthetic biologists who seek to implement these behaviors as building blocks for engineering robust, complex biological programs.

Here, we simulated all possible two- and three-component networks on random parameter sets, and assessed the resulting response profiles for degree of ultrasensitivity and bistability. Our strategy is partly inspired by a recent analysis of enzymatic networks that enable adaptation in bacteria [27]; however, in addition to studying networks with only enzyme components, we expanded our focus to include purely transcriptional networks and hybrid enzymatic/transcriptional networks which enabled us to quantify robustness with respect to both the function of each protein component in the network as well as the interactions among the components.

Our results reveal that network architecture and composition can have a dramatic impact on robustness in generating switch-like behavior. Specifically, compared to other compositional classes studied, hybrid networks are more robust in yielding ultrasensitive and bistable responses. Detailed analysis of network topologies suggests that the zero-order effect arising out of a simple enzymatic activation/inactivation system is a prevalent mechanism for generating robust ultrasensitivity, and hence can act as a building block for switch-like behavior. A global view of network topologies suggests strong clustering into a small number of recurring motifs. Finally, comparison with data from previous studies of natural and synthetic systems demonstrates concordance between these computational results and experimental observations, and highlights the utility of our analysis both as a discovery tool for studying how switching can arise in natural systems and as a design tool for engineering switch-like behavior in synthetic circuits.

## Results/Discussion

### Topology search scheme

To enumerate the network architectures that can give rise to switch-like behavior, we considered all possible topologies of two or three components, and assessed them for robustness in generating ultrasensitive and bistable responses. Although switch-like behavior can arise in networks having more than three components, restricting our scope to minimal networks makes the analysis more tractable and the results simpler to interpret. Moreover, many large networks can be reduced to minimal models without significant loss in the spectrum of behaviors observed [1], [28], [29].

**Figure 2:**
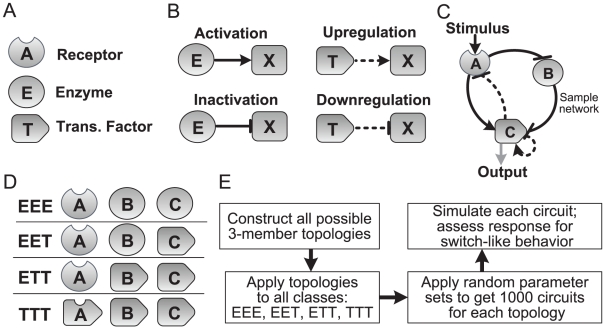
Topology search scheme. **A.** Each component is modeled as an enzyme or transcription factor. The input component *A* is modeled as a receptor to which the stimulus binds. **B.** Enzymatic components can catalyze the activation or inactivation of their targets, denoted as *X*. Transcriptional components can upregulate or inhibit the synthesis of the inactive forms of their targets. **C.** Sample network illustrating all possible interaction types. **D.** Four compositional classes were studied: EEE, in which *A*, *B*, *C*, are modeled as enzymes; TTT, in which each component is a transcription factor; and hybrid networks, in which only *C* is a transcription factor (EET) or both *B* and *C* are transcription factors (ETT). **E.** Overview of the topology search algorithm.

An overview of the search scheme is illustrated in Fig. 2. Each network topology considered consists of an input component, *A*, an output component, *C*, and if present, an additional component, *B*. The input component *A* is modeled as a receptor that is activated upon binding of the stimulus, *S*. The output component *C* is modeled as a downstream effector, and the level of active *C* is considered the response of the system. Allowing each component to activate, inhibit, or have no impact on the other two components and itself yields 3^9^ (19,683) distinct topologies. Within this set, approximately 3,700 topologies lack connections linking the input and output components, and are hence discarded.

Since activation and inhibition in biological systems can occur at both enzymatic and transcriptional levels, an important focus of this study is to compare the robustness in generating switch-like behavior arising out of enzyme and transcription components. Towards this goal, we studied four different categories of networks (Fig. 2D): enzyme-only, in which each component is modeled as an activating or inactivating enzyme (EEE); transcription-only, in which each component is modeled as a transcriptional activator or repressor (TTT); and two categories of hybrid networks, one with only *C* modeled as a transcriptional component (EET), and one with both *B* and *C* modeled as transcription components (ETT). While switch-like behavior can arise in networks belonging to other compositional classes, our study is focused on networks that can be directionally described as ‘outside-in’ signaling (i.e. networks that allow switch-like modulation of a downstream species, such as a master regulatory transcription factor that ushers in a phenotypic change, via an external stimulus).

In our analysis scheme, each component exists in an active form, which carries out the reactions specified by the network, and an inactive form, which only serves as substrate. Enzyme components act by catalyzing the inter-conversion of their targets. For instance, in the EET category, *B* is an enzyme, and an activation interaction from *B* to *C* denotes that *B* catalyzes the conversion of inactive *C* into active *C*; an inhibitory interaction would catalyze the opposite inter-conversion. Similarly, a positive interaction from *B* to *C* in the ETT category, in which *B* is a transcriptional component, denotes that *B* up-regulates the production of inactive *C*; an inhibitory interaction denotes *B*-mediated repression of the synthesis of *C*. Additionally, since enzymatic auto-regulation in signaling is not a common cellular behavior (e.g., there is a plethora of examples in which a kinase or phosphatase activates or inactivates another type of protein but not many instances in which an enzyme modifies its own species), only transcriptional components are allowed auto-regulatory loops, which reduces the number of network topologies considered for the EEE, EET, and ETT compositional classes. Irrespective of the topology, each component is modeled as being subject to basal synthesis and degradation and basal activation and inactivation by background components assumed to be constant.

A single network topology translates into a system of rate equations in which interactions among the three components are modeled using mass-action kinetics. Assignment of 10^3^ random parameter sets to the kinetic constants of this model yields 10^3^ different circuits having the same network architecture. Each circuit is simulated on a range of stimulus concentrations, and the resulting steady-state response information is assessed for switch-like behavior by two metrics: the Hill coefficient (*n_H_*), representing the degree of ultrasensitivity [8], and the relative drop in stimulus, or window (*W*) over which the system remains in the on state (Fig. 1). Hence, each network topology yields 10^3^ steady-state response plots. Parametric robustness in generating switch-like behavior is quantified by robustness scores representing the percent of plots exhibiting strong ultrasensitivity (*n_H_*>2), and bistability (*W*>5); for instance, a network that yields more ultrasensitive response profiles on random parameter sets than another is considered to be more robust in generating bistability. In addition to estimating *n_H_*, response steepness was also analyzed by computing the maximum local response coefficient (see Methods) [30]. Although both measures show good agreement (Fig. S1), since *n_H_* establishes a lower-bound on the steepness, it was used as the primary metric in assessing ultrasensitivity robustness. Our results also demonstrate that simulating 10^3^ random parameter sets for each network is sufficient for reliably estimating robustness scores (Fig. S2).

### Network composition influences robustness in generating switch-like behavior

In outside-in signaling systems, binding of a ligand to a receptor initiates a signaling cascade typically resulting in the activation of downstream transcription factors which can in turn alter the expression program of the cell, thereby ushering in phenotypic change [31], [32], [33], [34]. Hence, in ligand-activated systems, the switch-like nature of a response is most prominent at the transcriptional level, as is the case for instance in cell differentiation during development [10]. However, the actual circuitry enabling switch-like behavior may itself lie further upstream, and may be composed of transcription as well as enzyme components, which have fundamentally different properties and hence generate switch-like behavior via distinct mechanisms.

To assess the extent to which network composition influences robustness in generating switch-like behavior, we performed a global analysis of all network topologies across four compositional classes. Specifically, each network was simulated under the all-enzyme (EEE) compositional regime, and the resulting response profiles were used to compute a score quantifying the network's robustness in generating ultrasensitivity and bistability (as described above). The network was then re-simulated to obtain robustness scores under all-transcription (TTT) and hybrid (EET, ETT) regimes.

**Figure 3:**
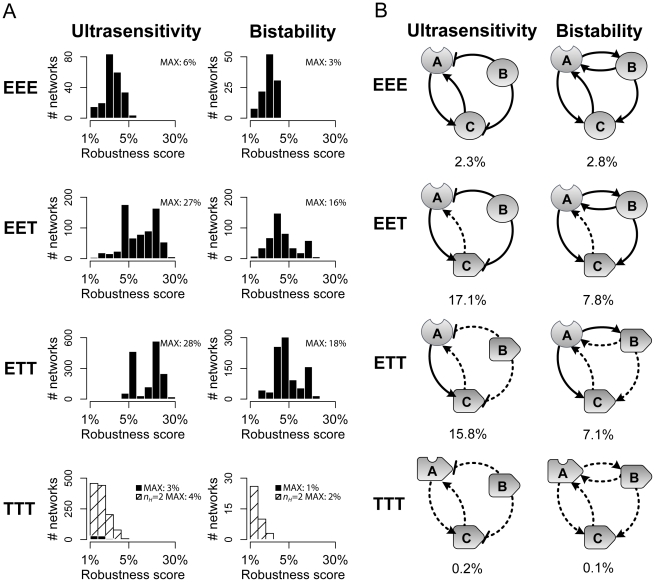
Robustness in switch-like behavior across compositional classes. **A.** All possible network topologies were constructed and simulated; response profiles were used to compute robustness scores for ultrasensitivity and bistability for each network topology. This process was repeated for each compositional class. Histograms depict the distribution of robustness scores for ultrasensitivity and bistability greater than 1% across all compositional classes; white bars with oblique lines in the TTT plots depict the distribution of robustness scores when each transcriptional interaction is modeled as being cooperative (*n_H_ = 2*). Histograms represent ultrasensitivity robustness scores for EEE (226 networks), EET (699), ETT (1511), TTT (84), TTT *n_H_* = 2 (1360) and bistability robustness scores for EEE (119 networks), EET (468), ETT (972), TTT (0), TTT *n_H_* = 2 (43). Networks achieving the highest robustness scores belong to the hybrid classes: the most robust networks in the ETT class achieve the highest scores for both ultrasensitivity and bistabiltiy, and the most robust networks in EET achieve comparably high scores. **B.** Ultrasensitivity and bistability robustness scores for two example topologies under different compositional classes; the same network topology can yield dramatically different robustness scores under different compositional classes.

First, across all compositional classes, a significantly larger number of networks demonstrated ultrasensitive behavior than bistable behavior (Fig. 3A), in line with the observation in biological systems that bistability is typically accompanied by ultrasensitivity [5], , but ultrasensitivity can also arise in the absence of bistability [8], [10], . Second, within a compositional class, a small proportion of networks exhibit switch-like behavior on a large percentage of random parameter sets. The highly skewed nature of robustness score distributions demonstrates that network architecture alone can impact robustness, and that a particular network's probability of generating switch-like behavior can be dramatically improved with rewiring, and without fine-tuning of kinetic constants such as those associated with binding or catalysis. Third, and most importantly, network composition strongly influences robustness in generating switch-like behavior. Compared to EEE and TTT classes, networks in the hybrid EET and ETT compositional classes yield ultrasensitive responses on a significantly larger proportion of parameter sets, with the most robust networks achieving ultrasensitivity robustness scores as high as 28%; in contrast, maximum ultrasensitivity robustness scores in the EEE and TTT classes are 6% and 3%, respectively. For bistability, maximum robustness scores for the EET and ETT compositional classes are approximately 16% and 18%, respectively, while scores for EEE and TTT classes are significantly lower at 3% and 1%, respectively (Fig. 3A). Our findings demonstrate that a particular network topology can yield markedly different robustness scores under different compositional regimes, and suggest that minimal networks composed of an enzyme input component, a transcription output component, and an additional enzyme or transcription regulatory node may be optimal for generating switch-like behavior.

### Transcription-only networks are suboptimal in generating switching, even with transcriptional cooperativity

Comparison of network topologies across different compositional classes reveals the unexpected result that purely transcriptional networks are markedly less robust in generating switch-like behavior. Despite the considerably enlarged set of networks analyzed—only transcription components were allowed self-regulatory links, yielding more possible topologies—the most robust TTT networks achieved dramatically lower robustness scores than those achieved by the most robust networks in the optimal EET and ETT categories.

In our analysis scheme, a transcriptional activation interaction represents the binding of a single transcription factor to a regulatory site, and is hence modeled as a linear reaction. However, a large number of transcription factors bind to DNA as dimers, and transcription initiation can itself be inherently cooperative [39]; both characteristics can directly introduce nonlinearity into a system, and therefore boost the probability of generating switch-like behavior [13], [40], [41]. To further investigate the impact of cooperativity arising out of multimerization and transcription initiation, we re-analyzed the entire set of networks in the TTT compositional class with all transcriptional interactions modeled as cooperative processes (*n_H_* = 2). As expected, robustness scores for both ultrasensitivity and bistability were enhanced, with the most robust networks generating ultrasensitive responses on 4%, and bistable responses on 2%, of parameter sets (Fig. 3A, slashed bars). However, despite including transcriptional cooperativity only in the TTT class (and not EET or ETT), the best networks in all other classes are still more robust than any network in the *n_H_* = 2 TTT class.

Our results suggest that, in terms of generating switch-like behavior, networks composed only of transcription components are inherently suboptimal relative to hybrid or all-enzyme compositional classes.

### Transcriptional feedback enhances switch-like behavior in hybrid networks

We now highlight some of the prevalent mechanisms contributing to the robustness differences between circuits in different compositional classes. In particular, we compare two network topologies in which a change in the identity of the output component *C* (i.e., either an enzyme or transcription component) leads to markedly different robustness scores for ultrasensitivity and bistability.

The network topology depicted in the left-hand column of Fig. 3B exhibits an ultrasensitive response on 2% of parameter sets in the EEE compositional context; however, when *C* is modeled as a transcription component, the robustness score for ultrasensitivity is dramatically higher, at 17%. Since *A* and *B* are modeled as enzymes under both EEE and EET regimes, the difference in robustness scores is entirely attributable to the feedback interaction from *C* to *A*, suggesting that transcriptional feedback enhances the probability of ultrasensitivity considerably more than activation feedback. To unravel the mechanisms contributing to the difference in robustness scores, we compared modules within this network to known models of ultrasensitivity.

We first examine the network that results when the feedback interaction from *C* to *A* is removed from the topology depicted in the left-hand column of Fig. 3B. Under both EEE and EET compositional classes, *A* acts as an enzyme activator for *C*, and *B* is effectively a background inactivator for both *A* and *C* (since there are no incoming links for *B*). When the total concentration (inactive and active) of *C* is much greater than those of active *A* and *B*, and the effective Michaelis constant (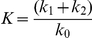, see Methods) values for activation and inactivation interactions are sufficiently small, enzymes *A* and *B* operate in a zero-order regime, which in turn causes the system to exhibit ultrasensitive activation of *C* [8]. Furthermore, transcriptional feedback from *C* to *A* can enhance existing ultrasensitivity or confer ultrasensitivity via an independent mechanism described in the next section.

Zero-order ultrasensitivity can also be generated or enhanced by transcriptional feedback merely via a concentration effect: feedback can significantly increase the amount of substrate, which may in turn enable the system to satisfy the conditions for zero-order ultrasensitivity. Hence, the presence of transcriptional feedback broadens the parameter sub-space in which the system yields an ultrasensitive response and boosts the overall probability of generating this behavior. Importantly, although the transcriptional feedback interaction does require minimal tuning to contribute to the overall robustness in generating ultrasensitivity, it does not hinder other mechanisms conferring this behavior.

Enzymatic activation feedback under the EEE compositional regime can give rise to strong ultrasensitivity [3]; however, in contrast to transcriptional feedback, activation feedback can also disrupt other interactions and thus narrow the parameter sub-space yielding ultrasensitive behavior. For instance, activation feedback can saturate active *A* (such that there are no more *A* molecules that can be converted into active *A*), thereby diminishing zero-order effects on *C*. Therefore, the network depicted in the left-hand column of Fig. 3B achieves a low robustness score, which changes marginally even when the feedback interaction is removed.

To understand mechanisms underlying differing robustness scores for bistability, we examined the network depicted in the right-hand column of Fig. 3B. This network generates a bistable response on 3% of parameter sets under the EEE compositional regime, and 8% when *C* is modeled as a transcription component (EET). This network contains two positive feedback interactions: between *B* and *A*, which is enzymatic under both EEE and EET regimes, and between *C* and *A*, which is transcriptional under EET and enzymatic under EEE. Removal of the feedback from *C* to *A* yields the same circuit under both EEE and EET, which achieves a robustness score of approximately 2%. In contrast, removal of the *B* to *A* feedback yields different circuits under EEE and EET, with robustness scores of 3% and 4%, respectively. Hence, while either feedback is sufficient for conferring bistability to the overall system, their combination leads to a significant increase in robustness under EET, but not under EEE.

A simple two-enzyme dual-activation system can exhibit bistability under certain parameter regimes [3]. In the EEE class, the network depicted in the right-hand column of Fig. 3B can achieve bistability via two separate enzymatic feedbacks. However, each feedback produces more active *A*, and can saturate it such that the addition of the second feedback (onto the same target *A*) has a diminished effect – since there is a limited quantity of inactive *A* that can be activated – and hence does not significantly broaden the parameter space for bistable behavior. In contrast, under EET, transcriptional feedback to *A* produces more inactive *A*, and hence does not hinder the enzymatic feedback from *B* to *A*. Although linear transcriptional feedback alone cannot generate bistability [38], [40], it can help confer this behavior in a network in which the activation interaction is independently ultrasensitive. Hence, under EET, the two feedbacks in the present network confer bistability via distinct mechanisms.

### Ultrasensitivity via linear transcriptional feedback and degradation

**Figure 4:**
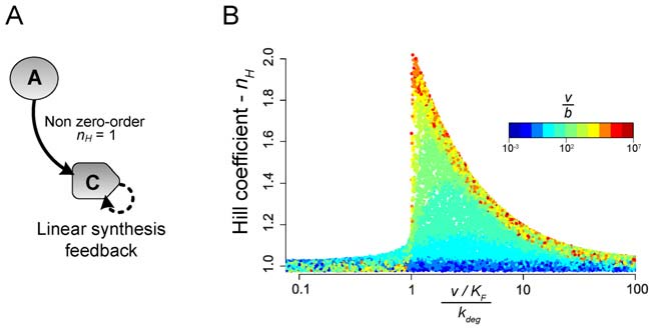
Ultrasensitivity via linear transcriptional feedback and degradation. A simple linear transcriptional feedback system can give rise to ultrasensitivity even in the absence of an inactivating enzyme. Note that this figure pertains to simulations on a minimal model different from the setup used for the topology search simulations (see Methods). **A.** In this system, the transcription factor *C* is activated by an enzyme, *A*. *C* is subject to basal synthesis and first-order degradation, but not to inactivation. **B.** The model was simulated on 10^6^ random parameter sets, and a random subset of the results was plotted. Each dot represents a separate simulation on a random parameter set, and the color of the dot denotes the value of the dimensionless ratio 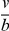 in that parameter set (where *b* is the basal synthesis rate and *v* is the maximal feedback synthesis rate). If 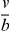 is sufficiently high, then the Hill coefficient reaches a maximum when the effective feedback synthesis rate constant 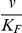 (where *K_F_* is the threshold concentration) is approximately equal to the degradation rate constant *k_deg_*.

Transcriptional feedback alone can give rise to modest ultrasensitivity via a mechanism distinct from zero-order ultrasensitivity. To investigate this phenomenon further, we separately modeled a simple system in which a transcription factor *C*, is activated by an enzyme *A*, and active *C* synthesizes more inactive *C* (Fig. 4A). *C* is synthesized and degraded via background processes, but unlike in our main topology search simulations, *C* is not subject to any inactivation process, which precludes the possibility of zero-order ultrasensitivity in any parameter regime. Parameter values for binding, dissociation, synthesis and degradation were varied and the resulting systems of ordinary differential equations were numerically integrated on a range of stimulus concentrations (see Methods for full model details). The resulting curves were then assessed for ultrasensitivity, and the results are summarized in Fig. 4B.

The results show that a simple transcriptional feedback system can generate responses with characteristic *n_H_* as high as 2, under certain parameter regimes. Interestingly, the extent of ultrasensitivity is independent of the explicit enzymatic binding, dissociation, and catalysis parameters, and instead is dependent on two dimensionless quantities. If the maximal feedback synthesis rate, *v*, is sufficiently greater than the basal synthesis rate, *b* (i.e., when
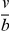
>>1), then *n_H_* reaches a maximum when the effective feedback synthesis rate constant 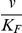 (where *K_F_* is the concentration of active *C* driving additional synthesis of inactive *C* at rate 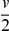) is approximately equal to the degradation rate constant *k_deg_* (i.e., when 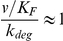). Hence, when feedback is strong, proper balance of feedback synthesis and degradation is sufficient to generate ultrasensitivity.

### Minimal architectures for generating ultrasensitivity

Having used our unbiased approach to discover pervasive, yet simple, interactions that augment the robustness of switch-like responses, we then took a design-centric view of our results to understand how these interactions could be combined to yield topologies exhibiting robust ultrasensitivity and bistability. Specifically, we focused on minimal networks (i.e., networks generating robust switch-like behavior with fewer interactions and components) for two main reasons. First, networks in biological systems arise via an evolutionary process, and since there is a cost associated with maintaining each interaction, natural selection is unlikely to maintain those interactions and components that do not contribute significantly towards enabling a necessary behavior (i.e., do not affect fitness). Second, minimal networks may suggest practical design strategies for engineering switch-like behavior in synthetic systems.

To identify minimal networks generating robust switch-like behavior, networks within each compositional class were ranked by the ultrasensitivity and bistability robustness scores, and only the top 100 networks in each category were retained. Next, a pruning step was performed. Briefly, within a particular category, each network was compared to every other network to determine if a proper subnetwork of this network having a higher robustness existed, or if this network's robustness score was within 15% of the maximum robustness score. If either was true, the network with more connections was removed from the list. This procedure filtered networks with excessive interactions, and made it easier to identify families of networks. The most robust networks after the filtering step are presented in rank order in Fig. S3.

A global view of the resulting topologies (Fig. S3) reveals strong consensus patterns and suggests that the set of robust, minimal networks readily clusters into a small number of families. Comparison of ultrasensitive and bistable networks within and across compositional classes reveals that networks with more interactions do not consistently rank higher than sparser networks, indicating that specific mechanisms conferring switch-like behavior cannot necessarily be combined to yield more robust networks, due to the possibility of interference. Despite this, a few simple motifs are particularly prevalent within a given compositional class (e.g., *A* activating *B*, which in turn activates *C* under EEE) and even across compositional classes (e.g., *A* activating *C*, which upregulates *A* under EET and ETT), indicating that such robust motifs can act as modular building blocks for conferring switch-like behavior to a system. In addition, the pruning procedure strikingly reduces each set of the 100 most robust networks to less than 20 networks in all but one compositional class, indicating that the set of networks generating robust switch-like behavior constitutes a very small fraction of the overall network space; below we discuss how this subspace reduces even further to a few distinct mechanisms.

**Figure 5:**
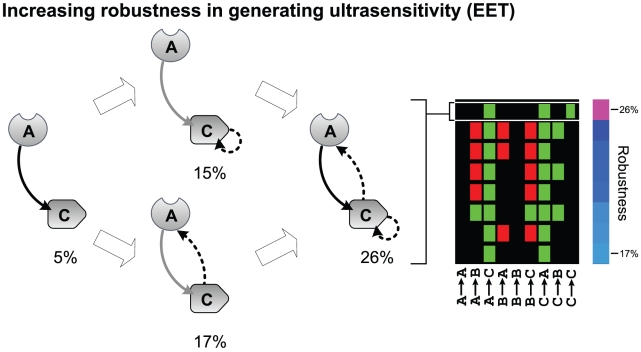
Minimal architecture for generating robust ultrasensitivity. Starting with a simple network, incremental addition of specific interactions significantly improves robustness in generating ultrasensitivity. The map to the right lists the eight most robust network topologies generating ultrasensitivity in the EET class, after pruning; positive, negative, and no interactions are depicted with green, red, and black, respectively.

The simplest network considered in our analysis, a two component topology with a positive interaction from *A* to *C*, yields an ultrasensitivity robustness score of approximately 5% under the EET compositional regime (Fig. 5). The ultrasensitivity exhibited by this circuit is entirely attributable to zero-order effects arising from the enzymatic cycle of induced activation of *A* and background inactivation. The addition of a transcriptional interaction from *C* to *A* yields a robustness score of 17%; strikingly, the *A*-to-*C*-to-*A* motif is present in all of the 100 most robust circuits in the EET class. An additional auto-regulatory transcriptional interaction onto *C* instead yields a robustness score of 15%. The combination of both *C*-to-*A* and *C*-to-*C* feedbacks yields a particularly high robustness score of 26%, making the dual-feedback circuit the most robust in the EET class after filtering. Together, the two feedbacks introduce independent non-interfering mechanisms for generating ultrasensitivity and enhance the probability of zero-order effects in the activation of *C* via a concentration effect. Thus, our analysis suggests that a simple network with two transcriptional feedbacks is among the most optimal configurations for generating ultrasensitivity.

Although networks in the all-enzyme EEE class yield significantly lower robustness scores, it is worth noting that the pruning procedure drastically trims the list of the 100 most robust networks in the EEE category to three very simple networks (Fig. S3). The most robust network, *A* activating *B*, which in turn activates *C*, represents a basic enzyme activation cascade. In the *A*-to-*B*-to-*C* network, ultrasensitivity can arise via two distinct mechanisms. First, the activation of *B* by *A* can be ultrasensitive if both *A* and the background inactivator for *B* behave in a zero-order manner. The ultrasensitivity can be further enhanced if the activation of *C* by *B* is similarly configured. Second, even in the absence of inactivating enzymes (and hence without zero-order effects), this cascade architecture itself can generate ultrasensitivity *de novo* [42].

### Minimal architectures for generating bistability

**Figure 6:**
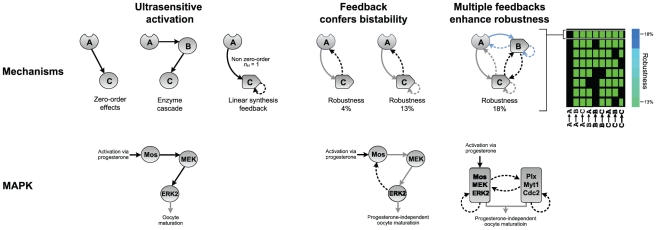
Coupling of ultrasensitive activation and positive synthesis feedback yields robust bistability. The upper row depicts molecular mechanisms derived from simulation results and the lower row depicts concordant examples in oocyte maturation. In our simulations, ultrasensitivity can arise via zero-order effects, enzyme cascading, and linear synthesis feedback. These motifs can yield bistability when coupled with positive synthesis feedback, and multiple feedbacks contribute to the robustness of this bistability. The map to the right lists the eight most robust network topologies generating bistability in the ETT class.

Examination of the most robust bistable networks in the ETT category (Fig. 6) reveals that although there is no obvious minimal motif conferring bistability, there is a clear bias towards multiple positive transcriptional feedback interactions. However, positive transcriptional feedback alone cannot confer bistability to a system, a point that is affirmed by the observation that the most robust networks in the transcriptional-only TTT category yield drastically lower scores. Closer inspection of the most robust networks reveals that in all of the top 100 networks, *A* activates *C*, which upregulates *A*. This simple hybrid motif of enzymatic activation and transcriptional feedback can yield bistability only if the activation step is independently ultrasensitive. In the space of networks considered in our analysis, bistability can arise via enzymatic activation and transcriptional feedback if the activation of *C* by *A* is ultrasensitive due to either zero-order effects or transcriptional autoregulation of *C*. Under ETT, bistability can also arise due to analogous interactions between *A* and *B*.

Importantly, our results also suggest that adding multiple instances of the enzymatic activation and transcriptional feedback motif to a single system does not hinder existing interactions, and can hence boost the probability of exhibiting a bistable response. In contrast, mechanisms such as cross-antagonism do appear in our analysis but are not highly ranked because of their stringent balancing requirements and fragility to interference by other interactions. For instance, in the two-component ETT network in which *A* activates *C*, and *C* upregulates *A* and itself, around 15% of the parameter sets yield ultrasensitivity but not bistability. To further explore the impact of combining motifs, we duplicated the dual transcriptional feedback motif in the same network by adding analogous interactions between *A* and *B*, and simulated the expanded network on the parameter sets that yielded ultrasensitivity but not bistability for the single motif network (parameter values for the added *A-B*, *B-B*, and *B-A* interactions were set to be the same as those for the *A-C*, *C-C*, and *C-A* interactions, respectively). We found that the expanded network with the duplicated motif converted more than 80% of previously ultrasensitive-only responses into strongly bistable responses. Since *B* and *C* are not directly connected in the expanded topology, the enhanced robustness can be attributed to increased nonlinearity in the activation response of *A*. Introduction of additional upregulation interactions from *B* to *C*, and *C* to *B*, further boosts the overall robustness score from 13% to 18%; this dual upregulation motif can confer bistability to circuits that exhibit only ultrasensitivity. While it is difficult to ascertain the exact contribution of each interaction in generating bistability as the network connectivity increases, our results point to the overarching principle that layering transcriptional feedback on an independently ultrasensitive activation interaction can act as a reusable building block for conferring bistability.

A noteworthy point about our results is that the robustness scores are bounded due in part to circuits which are otherwise bistable, but yield responses in which the ratio of maximum response to baseline response is low; this can arise in circuits with multiple positive feedbacks, for which basal activation alone is sufficient to switch the system into the on state. However, since our study is primarily focused on networks that can be modulated via an external stimulus, only responses that exhibit ≥10-fold increase in active *C* were considered.

### Comparison with networks in biological systems

Network families suggested by our analysis exhibit strong resemblance to circuits that have been previously shown to exhibit switch-like behavior in natural systems, and here we discuss a few striking examples of simple, elegant circuits that robustly regulate critical cellular decision-making.

**Figure 7:**
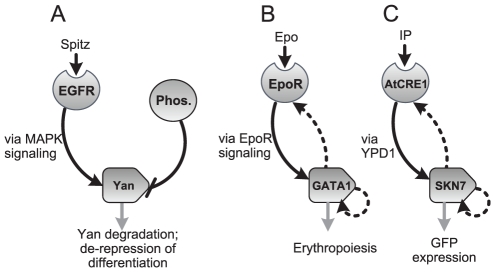
Comparison with natural and synthetic systems. **A.** Yan is a critical regulator of differentiation pathways in development, and generates ultrasensitivity via zero-order effects. **B.** The EpoR/GATA1 receptor/transcription factor pair can generate ultrasensitivity critical to the regulation of commitment to the erythrocytic lineage; this network is architecturally the same as the highest ranking network depicted in Fig. 5. **C.** The synthetic AtCRE1/SKN7 hybrid network depicted exhibits robust switch-like behavior in yeast. This network is architecturally the same as those in Figs. 5 and 7B.

The *Drosophila* protein Yan is a transcriptional repressor that inhibits differentiation; specifically, in the embryo, ultrasensitivity in Yan phosphorylation enforces a sharp boundary separating developmental domains [43]. Binding of the ligand Spitz to the epidermal growth factor receptor (EGFR) leads to the graded activation of the mitogen-activated protein kinase (MAPK) pathway, and eventually results in the phosphorylation of Yan; Yan dephosphorylation can occur via a separate phosphatase (Fig. 7A) [10], [44]. Phosphorylation of Yan makes it a target for degradation and thus promotes differentiation. Systematic perturbation of the network demonstrated that its robust ultrasensitivity is attributable to zero-order effects arising from the high levels of Yan relative to the concentrations of the kinase and phosphatase acting on this substrate [10].

MAPK pathways include a core, three-step cascade, and comprise an evolutionarily conserved family that enables eukaryotic cells to respond to a diverse array of signals [14], [45]. Ultrasensitivity has been observed in MAPK cascades in several organisms, most notably in *Xenopus* (Fig. 6). Immature *Xenopus* oocytes can be induced into maturation by treatment with the hormone progesterone, which acts via the MAPK signaling cascade: binding of progesterone to its receptor leads to the accumulation of active Mos, which activates MEK, which in turn activates ERK2 (also known as p42 MAPK). Active ERK2 can then activate cyclin B-CDK1 complexes which bring about entry into M-phase, leading to maturation. The three-tier cascade of Mos, MEK, and ERK2 has been demonstrated to exhibit ultrasensitive activation of ERK2 [35], [46]. The architecture of this cascade is essentially the same as the topology in the EEE class that ranks first in terms of robustness in generating ultrasensitivity in our analysis. Although ultrasensitivity in MAPK network can arise via several mechanisms, including zero-order effects and multi-site activation, the cascading architecture itself can amplify existing ultrasensitivity [47] and even generate ultrasensitivity where none exists [42].

The ERK2 response to progesterone treatment is also bistable. Immature oocytes treated with progesterone proceed to maturation even after progesterone is subsequently removed from the environment. The bistability observed in this system is attributed to a positive feedback from ERK2 that leads to increased synthesis of Mos [5]. Cdc2, another major driver of oocyte maturation, is involved in a positive feedback loop with Cdc25, and is also connected to the ERK2 system via mutual positive feedback interactions [5]. While important differences exist, the oocyte maturation system architecturally resembles the family of most robustly bistable topologies in the ETT class, which can yield ultrasensitive activation of *B* and *C* via zero-order effects or transcriptional feedback. Robust bistability can be generated by layering positive feedback onto ultrasensitive activation motifs, with additional minor gains in robustness achieved with positive crosstalk between ultrasensitive nodes (i.e., *B* and *C*). Similarly, the oocyte maturation system can generate ultrasensitive activation via cascading and other mechanisms, with robust bistability being achieved by multiple positive feedback interactions.

Another example is the network linking the erythropoietin receptor (EpoR) to the transcription factor GATA1 (Fig. 7B); it exhibits strong ultrasensitivity and helps confer bistability to the circuit regulating commitment to the erythrocytic lineage [36]. Briefly, the binding of the cytokine erythropoietin (Epo) to EpoR triggers the activation of GATA1, which in turn leads to the initiation of a transcriptional program for erythropoiesis. This circuit contains two feedback loops, with GATA1 transcriptionally up-regulating both EpoR and itself; the EpoR-GATA1 architecture is essentially the same as that depicted in Fig. 5 and described in the previous section; it ranks first in robustness (26%) in generating ultrasensitivity and also exhibits strong bistability (13% robustness).

### Step-wise dissection of a synthetic circuit

Networks achieving high robustness scores for ultrasensitivity and bistability have increased probabilities of exhibiting switch-like behavior in multiple biological systems and contexts. Although properties of components and the encompassing environment can constrain the effective parameter space and hence alter the ranking, a global analysis of topologies that can generate a desired behavior can help eliminate poor design choices and accelerate the implementation of synthetic circuits. We now highlight a few relevant findings from a separate study by our group which focused on the construction of a circuit exhibiting strong switch-like behavior [48], and we discuss how the topology search method can serve as an effective design tool for synthetic biology.

The synthetic *Saccharomyces cerevisiae* circuit depicted in Fig. 7C consists of the heterologously expressed *Arabidopsis thaliana* receptor CRE1 (AtCRE1), the endogenous SKN7 transcription factor, and GFP as a reporter, and is topologically the same as the ones presented in Figs. 5 and 7B. Binding of the cytokinin isopentenyladenine (IP) to yeast-expressed AtCRE1 has previously been shown to activate endogenous SKN7 [49], [50]. In our circuit, active SKN7 was synthetically wired to up-regulate the transcription of itself, AtCRE1, and the reporter GFP. To assess the contributions of specific topological connections in generating ultrasensitivity with respect to IP stimulus, the circuit was implemented in yeast with and without the feedback interactions. In the absence of feedback, the underlying circuit exhibits weak ultrasensitivity (*n_H_*≈2). Addition of receptor feedback does not impact ultrasensitivity regardless of promoter strength; since the total concentration of SKN7 is low, initial activation saturates active SKN7 levels before the feedback interaction can take effect. Autoregulation of SKN7 alone does non-trivially augment the ultrasensitivity (*n_H_*≈4); this enhancement arising from the increased concentration of SKN7 can be attributed to the non-linearity introduced by autoregulation (Fig. 4) and possibly to more pronounced zero-order effects if endogenous enzymes inactivate this transcription factor (Fig. 7A). The complete circuit with both feedback interactions exhibits extremely strong ultrasensitivity (*n_H_*≈20) and reasonable bistability (*W*≈2–3) in response to IP, which is in agreement with our predictions.

The primary objective of this study was to obtain a high-level architectural view of the network topologies yielding robust ultrasensitivity and bistability. To keep the simulations and subsequent analyses tractable, we employed simplifying assumptions which may affect interpretation of our results. First, for protein synthesis, transcription and translation processes were lumped into a single expression which may mask additional dynamics in the case of long-lived mRNA. Second, in our analysis scheme, transcriptional components upregulate the inactive form of their target species, and we find that this type of interaction alone in the TTT class is far less robust in yielding switch-like behavior; however, in some biological systems, transcription factors can effectively act as enzymes by interacting with other co-activators and co-repressors, and this can increase their ability to yield switch-like behavior. Third, we used simple thresholds for identifying responses as ultrasensitive (*n_H_*>2) and bistable (*W*>5), and did not focus on the extent of ultrasensitivity or bistability, which may be important in certain biological contexts; however, our general conclusions are not dependent on these specific filtering thresholds.

In conclusion, our analysis shows that although a large number of network topologies exhibit switch-like behavior, only a small fraction of the topologies can be expected to yield ultrasensitive and bistable responses in the context of a noisy and evolving environment. Network motifs generating robust ultrasensitive and bistable responses can help identify circuits with such properties in natural systems and can also suggest design strategies for synthetic implementation of switching behavior.

## Methods

### Network construction and modeling

The overall topology search scheme is based in part on a previously described method [27]. All possible two- and three-component topologies were constructed, with stimulus and active *C* considered the input and the response, respectively, for steady-state characterization (Fig. 1); networks lacking reachability from *A* to *C* were discarded. Depending on the compositional class analyzed, network components (*A*, *B*, *C*) were modeled as either enzymes or transcription factors. All components exist in two forms, inactive and active, which can be either free or bound to another species as part of a complex. Only active forms, denoted with an asterisk, carry out reactions. All species are subject to basal synthesis and degradation, as well as activation and inactivation by background components. For instance, accounting for background reactions leads to the following rate equations for *C* and C*:
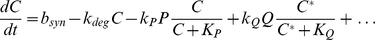
(1)

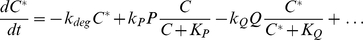
(2)where *P* and *Q* are the background activating and inactivating enzymes, respectively. Enzymatic interactions among main species were modeled using mass-action kinetics; for instance, here active enzyme B* binds to inactive *C*, forming a complex, *W*, which can either dissociate or catalyze the activation of *C* into C*:
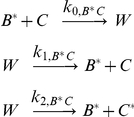
This set of interactions, modeled explicitly by law of mass action, yields the following terms in the relevant rate equations:
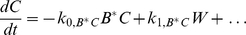
(3)

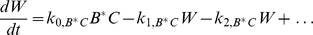
(4)

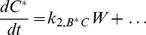
(5)

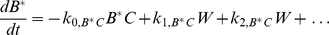
(6)Inactivation interactions are handled similarly, except that the intermediate complex consists of two active species; for instance, B* can inactivate C* by binding to it and releasing *C* after catalysis. (For this set of reactions describing the activation of *C* into C*, the effective Michaelis constant is 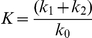.)

The stimulus for the system, *S*, binds to the receptor, *A*, in the form of a ligand:
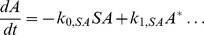
(7)

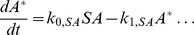
(8)The interaction between *A* and *S* is in addition to any interactions between *A* and other components, and background processes that act on all components, modeled by terms analogous to the ones depicted in equations 1–6. Collectively, interactions involving *A* represent two distinct biological mechanisms. The ligand-mediated activation of *A* represents a phosphorylation or other modification event immediately downstream; such a modification can also occur without involvement of the ligand, in which case this biological mechanism is modeled using enzymatic reactions.

Transcriptional interactions result in the upregulation of the inactive form of the target component; for instance, here active transcription factor B* upregulates inactive *C*:
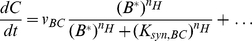
(9)A transcriptional Hill coefficient value of *n_H_* = 1 was used for all simulations, except for the re-simulation of circuits in the TTT class where *n_H_* = 2 was used, as described in Results/Discussion. Transcriptional inhibition is modeled as a competitive inhibition interaction; for instance, here A* inhibits the upregulation of *C* by B*:
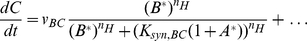
(10)A scheme similar to Latin hypercube sampling [51] was used to generate 10^3^ random parameter sets, with non-dimensionalized interaction parameter values (details given in Table S1) selected at uniform intervals on a logarithmic scale: *k*_0_∼(10^2^,10^3^); *k*_1_∼(10^0^,10^4^); *k*_2_, *k_P_*, *k_Q_*∼(10^1^,10^5^); *K_syn_*∼(10^−1^,10^1^); *K_P_*, *K_Q_*∼(10^−3^,10^1^); *v*∼(10^−3^,10^1^). Application of parameter sets yielded 10^3^ circuits for each network. Except where noted, the following parameters were held constant: *b_syn_* = 0.01, *k_deg_* = 0.01, *P* = 0.01, *Q* = 0.1.

### Simulation and assessment of switch-like behavior

Each naïve circuit was simulated to steady-state on a range of stimulus concentrations; levels of *A*, *B*, and *C* at the highest stimulus concentration were recorded and used as initial levels in another round of simulations to assess bistability. For ultrasensitivity, the stimulus levels at which the output reaches 10% and 90% were used to estimate *n_H_* (Fig. 1A) [8] and the following formula was used to estimate the maximum local response coefficient [30]:
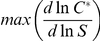
The forward and backward response profiles were used to estimate *W* (Fig. 1B); to be considered part of the bistable window of a response, the ratio of active C* in the forward and backward solves at a particular stimulus concentration had to be at least 5 and the difference had to be greater than 0.1. Activation responses not positively correlated with the stimulus or exhibiting less than a ten-fold increase from basal levels were not assessed for ultrasensitivity or bistability.

### Transcriptional feedback model

The separate transcriptional feedback system described in the text and presented in Fig. 4 was modeled as follows. *A* is an enzyme that catalyzes the conversion of *C* into C*, with the complex *Y* as an intermediate species. All species, *C*, *Y*, and C* are subject to first-order degradation. However, there is no inactivating enzyme, and hence zero-order ultrasensitivity cannot arise.
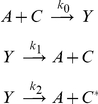


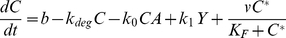
(11)

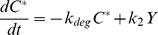
(12)

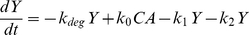
(13)


## Supporting Information

Figure S1 **Maximum local response coefficient correlates with estimated Hill coefficient** ***n_H_*** **.** In addition to estimating *n_H_*, the maximum local response coefficient was also computed for each network. This plot shows how the two metrics compare for all simulations of the double-feedback network topology under EET depicted in Fig. 5.
(EPS)

Figure S2 **Robustness scores converge in 10^3^ simulated parameter sets.** The double-feedback network depicted in Fig. 5 was simulated on 10^3^ parameter sets, for 100 runs. The histogram shows the distribution of robustness scores obtained.
(EPS)

Figure S3 **Network topologies ranked by robustness in generating ultrasensitivity and bistability.** Networks within each compositional class were ranked by ultrasensitivity and bistability robustness scores. Only network topologies ranking in the top 100 robust networks in their respective compositional classes were included. Networks with additional, non-contributing interactions were filtered from the list as described in the main text.
(EPS)

Table S1 **Parameter ranges and non-dimensionalization.**
(PDF)
