# Supplementary material for: Robust Network Topologies for Generating Switch-Like Cellular Responses
Source: PLoS Comput Biol. 2011 Jun 23;7(6):e1002085. doi: 10.1371/journal.pcbi.1002085 (PMC3121696; doi:10.1371/journal.pcbi.1002085)
Supplement: Table S1 — Parameter ranges and non-dimensionalization. (PDF) [file pcbi.1002085.s004.pdf]

Table S1: Parameter ranges and non-dimensionalization

Parameter	Description	Biological range		Scaling parameter			Non-dimensional
b_{syn}	basal synthesis rate	10^{-12}	Ms^{-1}	$\alpha\beta$	10^{10}	$M^{-1}s$	0.01
k_{deg}	basal degradation rate constant	10^{-5}	s^{-1}	α	10^{3}	s	0.01
K_P	basal activation Michaelis constant	$10^{-10}\cdots10^{-6}$	M	β	10^{7}	M^{-1}	$10^{-3}\cdots10^{1}$
k_P	basal activation catalytic rate constant	$10^{-2}\cdots10^{2}$	s^{-1}	α	10^{3}	s	$10^{1}\cdots10^{5}$
K_Q	basal inactivation Michaelis constant	$10^{-10}\cdots10^{-6}$	M	β	10^{7}	M^{-1}	$10^{-3}\cdots10^{1}$
k_Q	basal inactivation catalytic rate constant	$10^{-2}\cdots10^{2}$	s^{-1}	α	10^{3}	s	$10^{1}\cdots10^{5}$
k_0	complex association rate constant	$10^{6}\cdots10^{7}$	$M^{-1}s^{-1}$	$\frac{\alpha}{\beta}$	10^{-4}	Ms	$10^{2}\cdots10^{3}$
k_1	complex dissociation rate constant	$10^{-3}\cdots10^{1}$	s^{-1}	α	10^{3}	s	$10^{0}\cdots10^{4}$
k_2	catalytic rate constant	$10^{-2}\cdots10^{2}$	s^{-1}	α	10^{3}	s	$10^{1}\cdots10^{5}$
v	maximal transcription rate	$10^{-13}\cdots10^{-9}$	Ms^{-1}	$\alpha\beta$	10^{10}	$M^{-1}s$	$10^{-3}\cdots10^{1}$
K_{syn}	conc. at which transcription rate is $\frac{v}{2}$	$10^{-8}\cdots10^{-6}$	M	β	10^{7}	M^{-1}	$10^{-1}\cdots10^{1}$
P	basal activator concentration	10^{-9}	M	β	10^{7}	M^{-1}	0.01
Q	basal inactivator concentration	10^{-8}	M	β	10^{7}	M^{-1}	0.1

As an example, a simple association and dissociation reaction with species X, Y, and complex Z would be non-dimensionalized as follows.

$$X + Y \xrightarrow{k_0} Z$$
$$Z \xrightarrow{k_1} X + Y$$

$$Y_t = Y + Z$$
$$\hat{Y} = \frac{Y}{Y_t}$$
$$\hat{Z} = \frac{Z}{Y_t}$$

$$\frac{dZ}{dt} = k_0XY - k_1Z$$
$$\frac{d\hat{Z}}{dt} = k_0X\hat{Y} - k_1\hat{Z}$$
$$\alpha\frac{d\hat{Z}}{dt} = \alpha k_0X\hat{Y} - \alpha k_1\hat{Z}$$
$$\alpha\frac{d\hat{Z}}{dt} = \frac{\alpha k_0}{\beta}(\beta X)\hat{Y} - \alpha k_1\hat{Z}$$

Here, k_0 is multiplied by the scaling parameter $\frac{\alpha}{\beta}$ to obtain a non-dimensional k_0 and, similarly, k_1 is multiplied by α to obtain a non-dimensional k_1 (where $\alpha = 10^3s$ and $\beta = 10^7M^{-1}$ are constants). Repeating this non-dimensionalization procedure on the entire set of model equations yields the list of scaling parameters and non-dimensional parameter ranges in the table above.
